# Climate change and the potential effects on maternal and pregnancy outcomes: an assessment of the most vulnerable – the mother, fetus, and newborn child

**DOI:** 10.3402/gha.v6i0.19538

**Published:** 2013-03-11

**Authors:** Charlotta Rylander, Jon Øyvind Odland, Torkjel Manning Sandanger

**Affiliations:** 1 Department of Community Medicine, University of Tromsø, Tromsø, Norway; 2 Norwegian Institute for Air Research, Fram Research Centre, Tromsø, Norway

**Keywords:** climate change, reproductive health, pregnancy outcomes

## Abstract

In 2007, the Intergovernmental Panel on Climate Change (IPCC) presented a large amount of evidence about global warming and the impact of human activities on global climate change. The Lancet Commission have identified a number of ways in which climate change can influence human health: lack of food and safe drinking water, poor sanitation, population migration, changing disease patterns and morbidity, more frequent extreme weather events, and lack of shelter. Pregnant women, the developing fetus, and young children are considered the most vulnerable members of our species and are already marginalized in many countries. Therefore, they may have increased sensitivity to the effects of climate change. Published literature in the fields of climate change, human health, tropical diseases, and direct heat exposure were assessed through the regular search engines. This article demonstrates that climate change will increase the risk of infant and maternal mortality, birth complications, and poorer reproductive health, especially in tropical, developing countries. Thus, climate change will have a substantial impact on the health and survival of the next generation among already challenged populations. There is limited knowledge regarding which regions will be most heavily affected. Research efforts are therefore required to identify the most vulnerable populations, fill knowledge gaps, and coordinate efforts to reduce negative health consequences. The effects of malnutrition, infectious diseases, environmental problems, and direct heat exposure on maternal health outcomes will lead to severe health risks for mothers and children. Increased focus on antenatal care is recommended to prevent worsening maternal health and perinatal mortality and morbidity. Interventions to reduce the negative health impacts caused by climate change are also crucial. Every effort should be made to develop and maintain good antenatal care during extreme life conditions as a result of climate change.

In 2007, the Intergovernmental Panel on Climate Change (IPCC) presented a large amount of evidence about global warming and the impact of human activities on global climate (1). Global mean temperatures and ocean temperatures have increased over the last century, especially after the industrial revolution (1). The Antarctic ice cap is becoming thinner and the Arctic sea ice extent is declining. During the 20th century, global sea levels heightened. The frequency of extreme weather events, such as droughts and heavy precipitation, has increased at least over the last 30 years. At the same time, the concentration of the atmospheric ‘greenhouse gases’, carbon dioxide (CO_2_) and methane (CH_4_), have reached their highest levels for 650,000 years. The use of fossil fuels, land use, and agricultural activities are responsible for the increased concentrations of greenhouse gases over the last 250 years. IPCC concluded that it is extremely unlikely that the changes in global climate could have occurred without human activities (1). From 1910 until 2007, the global average temperature has increased by 0.74°C. The largest increase has occurred after the 1970s (2). A safe threshold of <2°C increase in global average temperature compared to preindustrial temperatures has been proposed (2), as the effects of climate change then would be manageable. Future prediction models and observations of ongoing trends indicate that the global average temperature is currently rising between 0.15 and 0.3°C per decade, which implies that the safe threshold will be exceeded during this century (1).

The effects of global warming on the local climate have varied between regions, countries, and specific locations (3). The predicted increase in temperature will be largest in the Arctic region. However, as the temperatures in these areas are originally low, the human health impact associated with a hot climate will most likely be small. In urban areas in the tropical region, local hot spots occur due to the ‘urban heat island effect’ (4) and concurrent poor air quality adds another risk factor to the impact of climate change on human health (5). Precipitation will vary in frequency, amount, intensity, and type with some areas getting drier and other areas getting wetter. Increasing humidity in hot areas adds to the heat stress and health risks (6).

All species on the Earth will be affected by climate change. Humans are no exception. Costello et al. (2) stated that ‘Climate change is the biggest global health threat of the 21st century’. The populations in developing countries in tropical areas are likely to suffer most from climate change due to poverty, poor sanitation, poor population health status, high population density, poor health care systems, and political instability, that is, limited government ability to cope with external crisis. The ability of health care systems to respond to an increase in the burden of climate-sensitive outcomes will play a critical role in determining the health impact of climate change.

The Lancet Commission have identified a number of ways in which climate change can influence human health: lack of food and safe drinking water, poor sanitation, population migration, changing disease pattern and morbidity, more frequent extreme weather events, and lack of shelter (2). Excessive heat exposure has also been identified as an emerging risk for human health by others (7). Pregnant women, the developing fetus, and young children are considered the most sensitive members of our species and are, in addition, already marginalized in many countries. They will therefore be most vulnerable to the environmental effects of climate change. A number of reviews have recently addressed the issue of health effects related to climate change, but there has been no analysis of maternal health, pregnancy outcome, and perinatal child health threats. Exposure to the effects of climate change at the early stage of development can cause immediate harm or damage that becomes evident later in life, resulting in lasting effects over a lifetime and even over generations (8). Thus, the aim of this article is to summarize how climate change can affect pregnant women and newborn children through some of the above-identified factors. The authors have recently written a comprehensive review of climate change and environmental impacts on maternal and newborn health, concluding that there is a need for intervention at many levels to reduce contaminant exposure and protect the future of the unborn and the newborn children (9).

## Maternal health and the millennium development goals

The World Health Organization (WHO) refers to maternal health as the health of women during pregnancy, childbirth, and the postpartum period. Each year, approximately 536,000 women die as a result of complications during pregnancy, childbirth, or the 6 weeks following delivery (10). Most women die from blood loss or hypertension, which accounts for half of all maternal deaths (the United Nations Millennium Development Goals; UN MDGs). Other indirect causes, such as malaria, HIV/AIDS, and heart diseases, account for 18% of all maternal deaths, whereas complications during childbirth accounts for 11%. Most maternal deaths are avoidable, and 99% of all maternal deaths related to childbearing or giving birth occur in developing countries (2).

In September 2000, the world leaders agreed on working together to achieve the eight MDGs of UN, all of them with the general aim to reduce extreme poverty, and improve education and health by 2015. The goal of MDG 5 is to improve maternal health by reducing the maternal mortality ratio by 75% and achieve universal access to reproductive health. The goal of MDG 4 is to reduce child mortality by two-thirds by 2015. Pneumonia, diarrhea, and malaria are three of the most common causes of death among children in developing countries under the age of five.

Climate change is considered one of the biggest threats to achieving the MDG for maternal health, clearly emphasizing the need for better and geographically more specific knowledge of climate change and maternal health (10).

Good maternal health is crucial for a healthy birth outcome and a healthy gestational period. In a recent review by Bhutta et al. (11), a clear link was established among reproductive health, maternal health, and perinatal birth outcome. Almost 3 million neonatal deaths and stillbirths yearly are attributable to maternal health conditions, such as poor nutritional status, deprived living environment, and infectious diseases (12, 13).

## Risk factors for maternal health and pregnancy outcome

The normal, frequent, or expectable temporary side effects of pregnancy are varied and complex, due to the physiological changes related to the significant hormonal changes (see Table 1). The normal side effects also keep changing throughout the pregnancy. General complications during the different stages of pregnancy are visualized in Table 2.

**Table 1:** Examples of clinical and physiological side effects of pregnancy due to occurrence of hormonal changes

First trimester	Second trimester	Third trimester
Exhaustion	Weight gain	Heartburn
Altered appetite	Dizziness	Indigestion
Nausea and vomiting	Fluid retention	Constipation
Weight loss	Haemorrhoids	Dizziness
Abdominal cramps	Abdominal cramps	Swelling
Yeast infections	Yeast infections	Fluid retention
Headaches	Backache	Haemorrhoids
Breast pain	Headaches	Yeast infections
Pica	Difficult sleep pattern	Backache
	Pica	Headaches
	Leg cramps	Difficult sleep pattern
	Joint pain	Discomfort while sleeping
	Hair loss	Increased urination and incontinence
		Pica
		Swelling of joints
		Leg cramps
		Increasing blood pressure
		Hair loss
		Anaemia

**Table 2:** Selected maternal complications at different stages of pregnancy

First trimester	Second trimester	Third trimester
Spontaneous abortion	Spontaneous abortion	Premature contractions
Missed abortion	Premature contractions	Abruptio placentae
Dehydration		Dehydration
Hyperemesis gravidarum		Renal failure
		Premature delivery
		Pre-eclampsia
		HELLP syndrome*

* Hemolysis; elevated liver enzymes; low platelet count.

In low- and middle-income countries, a pregnant woman is likely to be active in physical work (paid work or work in the household) until shortly before the delivery (5). The extra burden of physical activity during the latter parts of the pregnancy is normally not associated with premature delivery or intrauterine growth restriction (IUGR), but recent studies point out a possible connection (5). In addition, the practical problems of working with a large uterus are likely underestimated. Of other pregnancy-related problems, one might mention problems in performing physical work, infections due to immune suppression, and hormonal mood changes, including normal post-partum depression, especially related to heavy work load and responsibilities at a vulnerable stage of life (14). All of these factors lead to increased vulnerability for changes in external temperature, which sometimes result in serious changes in core body temperature (7). Specific complications during pregnancy with a focus on climate factors are shown in Table 3.

**Table 3:** An overview of possible diseases and complications to maternal and newborn health related to climate change

Disease/complication
Spontaneous abortion
Premature contractions
Low birth weight
Premature delivery
Increased neonatal mortality
Dehydration
Renal failure
Vector-borne diseases (e.g. malaria and dengue)
Malnutrition and food insecurity
Diarrhea
Respiratory disease
Water scarcity
Exposures to toxic chemicals
Worsened poverty
Natural disasters
Population displacement

## Interlinked effects; extreme events; population migration; lack of food, water, and sanitation; and changing disease pattern

The potential effects of climate change on human health are not only complex but also closely related. The frequency of extreme weather events, such as droughts and heavy precipitation, has increased over the past 3 decades (1). Whether this is an effect of climate change or not is debated. It is evident that drought has resulted in massive livestock deaths, crop failure, and severe malnutrition. A recent study performed by the Division of Global Disease Detection and Emergency Response at Center of Disease Control and Prevention in 15 regions of Somalia revealed that the prevalence of global acute malnutrition exceeded 20% in all regions (13). The crude mortality ratio (CMR) for children under the age of five varied between 4.1 and 20.3 deaths/10,000/day in all regions. Several hundred thousand people have become refugees due to the drought, and it has been estimated that the world's largest refugee camp, Dadaab in Kenya, experiences more than 1,000 new arrivals each day (13).

Natural disasters or extreme weather events, such as droughts, have a large impact on human health by a number of primary and secondary factors. Crop failure, livestock mortality, and increased cereal prices will cause food shortage and malnutrition. In many regions all over the world, food is already scarce, and climate change is believed to further reduce the availability in populations that are already pressured. The situation in poor countries with on-going armed conflicts is already alarming, and the changes in food supply and food security add new problems to an already complicated situation. It has been estimated that the total population of food-insecure countries may increase to 6.8 billion or approximately 80% of the world population (15). The probability of the food being contaminated or of poor quality will also increase in areas already facing difficulties. It is evident that the shortage of food and malnutrition are going to be key issues with climate change.

Safe drinking water is essential for good health, yet it is still unavailable to more than 1 billion people worldwide (16). Natural disasters or extreme events will reduce the access to safe drinking water and proper sanitation, thereby increasing the risk of malnutrition, diarrhea, and cholera. The WHO has characterized these three health outcomes as being among the most climate-sensitive ones (17). The effects of these diseases are further confounded by undernutrition. Undernutrition, including stunting, poor fetal growth, and micronutrient deficiencies, is the underlying cause of at least 3.5 million deaths (18). In 2010, 317,534 cases of cholera in 48 different countries were reported to the WHO (18). Over the past 15 years, the African continent has reported the majority of cholera cases; however in 2010, only 36% occurred in Africa and 56% in the Americas (19). The trend shift is explained by the devastating earthquake in Haiti that led to a cholera outbreak in 2010, which also spread to the Dominican Republic (19). This situation clearly emphasizes that increased frequency of extreme events has a large impact on human health.

In addition, lack of safe drinking water and food will lead to migration of people, which in turn can create tense situations between ethnic groups. Several hundreds of millions of climate refugees are expected by 2050 due to droughts, natural disasters, sea level rise, lack of food and water, and so on. (2). Care for the pregnant mother will be considerably reduced or impossible to obtain in such situations. Increased migration will further increase the risk of the emergence of epidemics in new areas since the spreading and control of many tropical diseases has been linked to population movements (20).

## Climate-induced health risks for pregnant women and newborns

### Malnutrition, maternal health, and pregnancy outcome

A well-nourished woman is essential for a healthy gestation period. During pregnancy, the energy demand of women increases by approximately 20%, which also continues throughout the period of breastfeeding. Maternal undernutrition is defined as having a body mass index (BMI) less than 18.5 kg/m^2^. In most countries in sub-Saharan Africa, more than 20% of the women are classified as being malnourished (18). Malnutrition is also a large problem among women in many countries in South East Asia.

Underweight women are more likely to give birth to children suffering from IUGR/low birth weight (18), which is considered a risk factor for infant morbidity and infant mortality (21). Short stature is also a risk factor for birth complications; however, there is no association between low BMI and birth complications (18). Undernutrition is the leading cause for child morbidity and child mortality in sub-Saharan Africa (18), where 2.2 million deaths among children under the age of five and 1.3 million deaths among mothers are estimated to be attributable to undernutrition (18). Thirty-five percent of the disease burden among young children is estimated to be the result of undernutrition. Underweight children are also more susceptible to infectious diseases (malaria, pneumonia, diarrhea, etc.), and poor diet is associated with growth retardation and delayed mental development (22). In the short-term perspective, undernutrition among mother or child will increase the risk of morbidity and mortality during infancy, whereas the same condition could affect the intellectual and reproductive ability, the economic productivity, and increase the risk for metabolic disorders in a long-term perspective (21).

### Diarrhea, cholera, maternal health, and pregnancy outcome

Diarrheal disease, including cholera, is the third most common cause of death for the population in low-income countries and is responsible for approximately 1.8 million deaths each year (23). For children under the age of five, it is the second most common cause of death, after pneumonia (24). Diarrhea and cholera often lead to dehydration, which is a life-threatening condition. The most important clinical factor influencing the pregnancy outcome and the child's health condition in the neonatal period is the dehydration related to severe maternal diarrhea (25). Climate change is predicted to increase the risk and severity of diarrheal diseases, and it has been suggested that the disease may increase by millions of cases with each degree of increase in ambient temperature above normal (26).

### Vector-borne diseases, maternal health, and pregnancy outcome

Increased temperatures are expected to increase transmission and spreading of vector-borne diseases by increasing mosquito density in some areas and increase in replication rate and bite frequency of mosquitoes (2). Costello et al. (2) argue that malaria, dengue fever and tick-borne encephalitis will become more widespread and that humans not currently infected and with no, or poor immunity, will be at risk in the future. Also schistosomiasis may infect more people in the future (16).

#### Malaria

Malaria is currently the most deadly vector-borne disease in the world. In 2008, 247 million cases of Malaria were reported and about 1 million deaths (27). Most of them were African children under the age of five (28). Malaria actually accounts for 20% of all childhood deaths in Africa (27). The effect of climate change on Malaria has been debated. Costello et al. expect an increasing number of cases (2). According to Reiter (29), climate change may alter the prevalence and incidence of Malaria, but increased temperature is not the only cause, rather a secondary effect of climate change in the form of changes in population density, precipitation pattern, and ecological changes of both humans and the vectors have a bearing on the incidence of malaria.

Approximately 125 million pregnant women are infected by malaria each year (30). Malaria is known to cause 75,000–200,000 infant deaths yearly in sub-Saharan Africa, and a large proportion of maternal deaths (24–37%) during pregnancy are attributable to Malaria in several African countries (31). In endemic areas, Malaria can cause placental malaria and severe malaria anemia (31). Maternal malaria infection might lead to IUGR and prematurity among newborn, which in turn may lead to low birth weight (14, 32).

Several studies from sub-Saharan Africa (Gabon, Cameroon, and Tanzania) have identified a link between the risk of malaria morbidity during infancy and a malaria-infected mother (33). In all of the above-mentioned studies, the mothers had malaria-infected placentas. Placental malaria has also been associated with low birth weight (34) and *in utero* malaria exposure has been related to increased risk of malaria infection or anemia during childhood (35). In a recent randomized controlled trial of 1,030 pregnant Mozambican women, there was an increased risk of infant mortality if the mother had acute malaria-infected placenta (33). The risk of infant mortality was also increased by low birth weight. In addition, there was an increased risk of clinical malaria during infancy if the mother had clinical malaria during pregnancy or had acute malaria-infected placenta. There are good indications that the use of insecticide treated bed nets during pregnancy is effective in reducing fetal loss (8).

#### Dengue fever

Dengue fever is one of the most common mosquito-borne infections in the world with around 100 million cases each year. WHO states that presently dengue fever and dengue hemorrhagic fever are the fastest growing vector-borne diseases (28). With increasing temperatures, the geographical area of dengue transmission is expected to increase (as a primary or secondary cause of climate change) and approximately 5–6 billion people will be at risk of the disease by the end of the century (16). Due to factors like population density and lack of prevention strategies, dengue fever is a large health problem in many developing, tropical countries.

There are a number of adverse health effects and pregnancy outcomes that are suspected to be linked to maternal dengue infection during pregnancy. These include vertical transmission to the fetus, preterm birth, low birth weight, pre-eclampsia and eclampsia, Caesarean delivery, and fetal/perinatal death and maternal death. In 2010, Pouliot et al. (36) published a systematic review on maternal dengue and pregnancy outcomes. The authors pointed out that it seems plausible that there is a risk of vertical transmission of dengue and that the risk increases with increasing severity of maternal dengue. The authors emphasized that little is known about the effects of maternal dengue and pregnancy outcomes.

#### Schistosomiasis (bilharzia)

Schistosomiasis, also commonly known as bilharzia, is a chronic disease caused by parasitic flatworms from the schistosome (37). There are five species of schistosome that infect humans; however, three are more common than the others: *Schistosoma mansoni, Schistosoma japonicum*, and *Schistosoma haematobium* (38). The schistosomes use humans as their final host and molluscs as their intermediate host, which is why the disease is also known as ‘snail fever’. Due to the route of transmission, people collecting water from rivers and streams and children that play in water are especially exposed to the disease.

According to the WHO (37), approximately 700 million people are currently at risk for schistosomiasis in 74 endemic countries and more than 200 million people are infected. Approximately 40 million women of childbearing age are estimated to be infected by the disease and 10 million pregnant women live with the disease (12). The majority of infected people (85%) live in sub-Saharan Africa. The WHO considers schistosomiasis as one of the three most devastating tropical disease.

Climate change will affect water resources and millions of people are estimated to become climate refugees. As a consequence, more people will lack access to safe drinking water and proper sanitation. This will facilitate the emergence of schistosomiasis, which will increase the risk of the disease becoming endemic in areas that currently have low endemicity. In Brazil and Eastern Africa, refugee movements and migrations have already demonstrated the spreading of schistosomiasis into areas that were previously not infected (20).

There are several case reports and case series that report the effect of schistosomiasis on pregnancy health and birth outcome. Unfortunately, there are few proper comparative studies which investigate the effects of schistosomiasis on pregnancy health, and it is therefore complicated to establish a causal relationship between the disease and suspected outcomes. Rodent models suggest that schistosomiasis has a strong negative impact on birth outcome. However, the disease is more strongly expressed in mice than in humans, and the results should therefore be interpreted with care (12). There are a few population-based studies on pregnant women. Ajanga et al. (39) identified a significant association between being heavily infected by S. *mansoni* and increased risk of anemia among 972 pregnant women. Anemia is a risk factor for low birth weight and maternal mortality, and low birth weight is in turn a risk factor for infant mortality (32). In a recent observational study of 99 pregnant women residing in the Philippines, maternal *S. japonicum* was associated with maternal, placental, and fetal inflammation, and placental inflammation was also associated with low birth weight (40).

### Extreme events and migration, maternal health, and pregnancy outcome

The birth outcomes of migrants have been assessed in a number of studies with conflicting findings (41, 42). It is however evident that people that are forced to resettle due to natural disasters or war are stressed due to traumatized experiences. In addition, they could have been travelling for months with variable access to safe drinking water and food. It has been shown that the spreading and control of many tropical diseases are linked to population movements and that the process is often exacerbated by poor medical service and poor sanitary conditions (20). The health status among migrants is therefore often poorer than the health status among people in the host country. Women and children that are already marginalized will be no exception. In a study of internally displaced people in western Kenya, children under the age of five had an almost three-fold risk of being hospitalized compared to children that were not internally displaced (43). For persons over the age of five, there was an increased risk of dying, in general, if being internally displaced and an increased risk of dying from HIV in particular (43).

Traumatic experiences will also affect the mental health of people. Perinatal common mental disorder (PCMD) is common among mothers in low-income countries (44), and it has been shown that postpartum depression is more common among immigrant mothers (45). Mothers with PCMD are more likely to beget a child suffering from diarrhea (46) or a child with low birth weight (47). Both conditions are risk factors for infant mortality.

In regards to maternal health, extreme events will, in addition to reducing access to safe drinking water and food and increasing the risk of infectious diseases, also affect the access to hospital and pregnancy care. Lack of access to proper pregnancy care will further increase the risk for complications during delivery and also increase the risk of maternal and infant mortality.

### Effects of direct heat exposure on maternal health and birth outcome

The effects of direct heat exposure imply the potential health effects of increased core body temperature due to increasing surrounding temperatures.

The physical exchange of heat between the human body and the surrounding air is essential for maintaining a stable core body temperature (37°C). If the air is hotter than the body, heat will be added to the body. Strong heat radiation that reaches the skin (e.g. via sun rays or from hot equipment) also adds heat to the body, depending on clothing or shading facilities (14). A person's capacity to reduce excessive heat by sweat evaporation to maintain core body temperature is strongly influenced by the surrounding temperature, humidity, wind, and clothing. The rate of sweat evaporation is reduced with increasing humidity and increased by air movement (providing the humidity is not greater than 80%). Therefore, heat stress indicators incorporate humidity and wind speed, as well as temperature.

If core body temperature exceeds 38**°** for several hours, heat exhaustion and reduced psychometric and motor capacity will occur. If core body temperature continues to rise (>39**°**), an unconscious condition may occur (7). During leisure time, people are able to adjust to ambient temperatures in many ways, for example, through spending time in the shade and indoors, drinking water, and by swimming. However, during working hours, many people in developing countries are not able to make these adjustments as they do hard physical labor in the sun or work in a factory with no air-conditioning (7).

With increasing temperatures due to climate change, the risk of heat shock will be largest in already hot countries and for people with physically demanding labor. In El Salvador and Nicaragua, many cases of serious chronic kidney disease are observed among young people working in the sugar industry (7). The workers spend 6–8 hours outdoors each day over several months. They often suffer from dehydration, which likely is the cause of kidney disease. In Southern India and Northern Vietnam, reduced productivity in factories is observed during the hot periods as the temperature may be above 40**°** because air-conditioning is not available in many manufactoring units (7).

Pregnant women are at particular risk of ‘over-heating’ (too high core body temperature) because of the hormonal situation at all stages of pregnancy (14). This increases the health risk for both mother and fetus (14). The newborn is especially sensitive to too high or too low temperature in the environment because of its limited temperature regulation capacity (5). Strand et al. have explored seasonal patterns of birth outcomes, such as low birth weight, preterm birth, and stillbirth, in relation to ambient temperature (48). The authors reviewed the epidemiological evidence on seasonality of birth outcomes and the impact of prenatal exposure to ambient temperature on birth outcomes. Most of the studies found peaks of preterm birth, stillbirth, and low birth weight in winter, summer or both, which indicates that the extremes of temperature may be a risk factor for poor birth outcome. Sheffield et al. (49) have assessed children's health and climate change in a global perspective. They concluded that heat-related health effects include diminished school performance, increased rates of pregnancy complications, and renal effects. The severity of these outcomes will vary by geographical region and socioeconomic status, further increasing health inequalities.

## Discussion and future perspectives

This article has focused on how climate change can influence maternal and pregnancy health through lack of food, safe drinking water, and proper sanitation; increased frequency of extreme weather events; migrations; changing patterns of disease and morbidity; and direct heat exposure. All of these factors are closely related and will interact. Increased frequency of extreme weather events will lead to malnutrition, cholera outbreaks, population movements, and spreading of diseases. Migration is often a result of armed conflicts between ethnic groups, and it adds additional risk for pregnant women and the unborn child. Lack of access to proper health care and increased core body temperatures due to hot ambient surroundings will further increase the health risks for the pregnant and delivering women. Specifically, the consequences for pregnant women and the unborn child can be substantial. Thus, global efforts to reduce the negative health effects of climate change should focus on maternal health.

Malnutrition and diarrheal diseases are already major causes of child mortality in tropical regions. Additional lack of food and safe drinking water as a result of climate change will further increase maternal and child mortality, especially in sub-Saharan Africa and in areas devastated by floods, droughts, earthquakes, and so on. An undernourished child is also more likely to contract other infections (e.g. pneumonia and malaria) that could be life-threatening. A significant increase of pregnant women in the Southern hemisphere contracting malaria, dengue fever, and schistosomiasis before the end of this century is expected, as a result of changing disease pattern and population migration. Malaria is already a major cause of maternal deaths during pregnancy and the death of children under the age of five (24). Increased incidence of malaria, dengue fever, and schistosomiasis will clearly increase the risk of begetting a child with low birth weight. The risk for birth complications and preterm deliveries will also increase. Thus, these diseases will lead to increased infant and maternal mortality, poorer reproductive health, and increased morbidity among young children.

Increased incidence of all the above-mentioned diseases will demand better health care systems and better access to medicines for hundreds of millions of children and women in the years to come. This will put an enormous pressure on future governments, and many countries will probably not be ready to handle these challenges. Climate change will therefore threaten the achievement of the MDG for maternal health (MDG 5) (10). In addition, climate change will also threaten the achievement of MDG 4 – to reduce child mortality.

This article shows that climate change will have large impacts on the health and survival of the next generation among the already challenged populations of the world. However, there is limited knowledge about which regions will be most heavily influenced and exactly what climatic changes will occur in the specific areas and what consequences they will have. Considerable research efforts are therefore required to identify the most vulnerable populations, fill knowledge gaps, and coordinate efforts to reduce the negative health consequences. The effects of cholera, malnutrition, malaria, dengue infection, schistosomiasis, and direct heat exposure on maternal health outcome are not well understood, even though there are strong indications that these conditions will lead to severe health risks for mother and child. An additional effect of the climate change is the changed behavior of environmental contaminants and their pathways into the human body through diet, inhalation, and dermal contact. This topic is thoroughly discussed by the authors in another article (9).

Both Costello and Homer (2, 10) argued that family planning, including strengthening of women's education, is the only way forward from this point. Empowering women will bring many positive additive effects, such as reduced poverty, reduced maternal and child mortality, and reduced population growth. In addition, it has been shown that the lack of appropriate antenatal care significantly increases the risk of perinatal mortality (10). Making good quality antenatal care available is in itself beneficial for maternal health outcome. It is also an intervention to reduce the negative health impacts of climate change. Every effort should therefore be made to maintain antenatal care during extreme conditions. Delivery assistance by educated birth attendants will be just as important to prevent maternal and neonatal mortality and morbidity associated with climate change, especially in developing countries.
